# PAI-1: A Major Player in the Vascular Dysfunction in Obstructive Sleep Apnea?

**DOI:** 10.3390/ijms23105516

**Published:** 2022-05-15

**Authors:** Mohammad Badran, David Gozal

**Affiliations:** 1 Department of Child Health and Child Health Research Institute, School of Medicine, University of Missouri, 400 N Keene St, Suite 010, Columbia, MO 65201, USA; mbadran@health.missouri.edu; 2 Department of Medical Pharmacology and Physiology, School of Medicine, University of Missouri, Columbia, MO 65201, USA

**Keywords:** obstructive sleep apnea, intermittent hypoxia, plasminogen activator inhibitor-1, endothelial dysfunction, atherosclerosis

## Abstract

Obstructive sleep apnea is a chronic and prevalent condition that is associated with endothelial dysfunction, atherosclerosis, and imposes excess overall cardiovascular risk and mortality. Despite its high prevalence and the susceptibility of CVD patients to OSA-mediated stressors, OSA is still under-recognized and untreated in cardiovascular practice. Moreover, conventional OSA treatments have yielded either controversial or disappointing results in terms of protection against CVD, prompting the need for the identification of additional mechanisms and associated adjuvant therapies. Plasminogen activator inhibitor-1 (PAI-1), the primary inhibitor of tissue-type plasminogen activator (tPA) and urinary-type plasminogen activator (uPA), is a key regulator of fibrinolysis and cell migration. Indeed, elevated PAI-1 expression is associated with major cardiovascular adverse events that have been attributed to its antifibrinolytic activity. However, extensive evidence indicates that PAI-1 can induce endothelial dysfunction and atherosclerosis through complex interactions within the vasculature in an antifibrinolytic-independent matter. Elevated PAI-1 levels have been reported in OSA patients. However, the impact of PAI-1 on OSA-induced CVD has not been addressed to date. Here, we provide a comprehensive review on the mechanisms by which OSA and its most detrimental perturbation, intermittent hypoxia (IH), can enhance the transcription of PAI-1. We also propose causal pathways by which PAI-1 can promote atherosclerosis in OSA, thereby identifying PAI-1 as a potential therapeutic target in OSA-induced CVD.

## 1. Introduction

Obstructive sleep apnea (OSA) is a chronic condition affecting up to one billion people worldwide [1]. OSA is defined as a sleep-breathing disorder that involves a decrease or complete cessation of airflow despite ongoing efforts to breathe due to a collapsed upper airway. This leads to partial reductions (hypopneas) and complete pauses (apneas) in breathing that usually last between 10 and 30 s, but some may persist longer. This can lead to abrupt reductions in blood oxygen saturation, with oxygen levels falling as much as 40% or more in severe cases [2]. As a result, several pathological mechanisms ensue such as intermittent hypoxia (IH), sleep fragmentation, episodic hypercapnia, and increased intrathoracic pressure swings [3,4,5]. Consequently, these processes can induce major changes in the autonomic nervous system balance with both increased tonic and reactive sympathetic activity along with parasympathetic withdrawal, disruption of the hypothalamic–pituitary–adrenal-axis, systemic and cellular oxidative stress, and inflammation, fibrosis, and accelerated cellular senescence, all of which resulted in neurocognitive deficits, endothelial dysfunction, hypertension, and atherosclerosis [6,7,8,9,10,11,12,13]. Predictably, OSA is considered as an independent risk factor for cardiovascular disease (CVD) including coronary artery disease (CAD), ischemic stroke, and myocardial infarction (MI) [14]. The majority of strokes and MIs seem to be prompted by atherothrombotic events along with compromised fibrinolytic activity, increasing the propensity for such events [15,16]. The fibrinolytic system is designed to cleave the insoluble polymeric network of fibrin from the vascular system to prevent clot overgrowth and vessel occlusion. Generally, plasminogen is activated by serine proteases plasminogen activators (PAs) including tissue-type PA (tPA) and urokinase-type PA (uPA) into plasmin, which in turn lyses the fibrin and other extracellular matrix components [17,18]. To prevent bleeding, plasminogen activator inhibitor-1 (PAI-1) is normally synthesized in equimolar amounts to PAs, forms a covalent bond with Pas, and stabilizes fibrin [19]. However, many processes including oxidative stress [20], inflammation [21], and fibrosis [22] can lead to elevated levels of PAI-1, which have been implicated in a multitude of diseases and conditions including CVD [23], cancer [24], metabolic disease [25], renal disease [26], behavioral and psychiatric conditions [27], and aging processes [28]. Furthermore, PAI-1 has been shown to induce endothelial dysfunction and atherosclerosis through antifibrinolytic-dependent mechanisms including inflammation [29], endothelial nitric oxide synthase (eNOS) inhibition [30], neointimal hyperplasia [31], and vascular senescence [28]. Despite the fact that PAI-1 levels are consistently elevated in OSA patients [32,33,34,35,36,37,38,39,40,41,42], and that OSA can trigger processes that can upregulate PAI-1 production, little to no attention has been given to PAI-1 as a biomarker or as a promoter of OSA-induced CVD in clinical practice. Here, we will summarize the mechanisms involved in upregulating PAI-1, the pathological role of PAI-1 in CVD, and underline the mechanisms by which OSA could upregulate PAI-1, thus, highlighting PAI-1 as a potential therapeutic target in OSA-induced CVD. Finally, we will discuss some of the therapeutic approaches to reduce PAI-1 levels, which may hold promise as adjuvant therapies in OSA since existing treatments (e.g., continuous positive airway pressure (CPAP)) appear to be ineffective in reversing or mitigating the frequency and severity of cardiovascular events in OSA patients [43,44].

## 2. PAI-1 Sources, Structure, and Function

PAI-1 can be synthetized by numerous types of cells including platelets, macrophages, adipocytes, hepatocytes, vascular smooth muscle cells, endothelial cells, and others [45,46,47]. Approximately 10% of the PAI-1 produced circulates in the blood or is deposited in the subendothelial matrix, while the rest is retained in platelets [48,49]. Platelets can de novo synthesize PAI-1 despite lacking nuclei through activated PAI-1 mRNA, with the synthesis rates being increased upon platelet activation [50]. The circulating PAI-1 fraction exists in its active conformation at levels of 5–50 ng/mL with large intra- and inter-personal variability, while platelet PAI-1 concentrations can reach up to 300 ng/mL with 50% shown to be biologically active [48,51,52]. Ultimately, PAI-1 plasma levels are increased under numerous pathological conditions [53]. The structure and function of PAI-1 have been extensively reviewed previously [25,54,55]. Briefly, PAI-1 is a single chain molecule with two interactive domains including a surface-exposed reactive center loop (RCL) that presents as a substrate peptide becoming the primary site for uPA/tPA binding, and a flexible joint region with helices D, E, and F that bind to vitronectin and stabilize PAI-1 in its active form while enhancing its binding affinity to uPA/tPA 200-fold [24,56,57,58,59,60,61]. PAI-1 exists in three distinct structurally and functionally distinct conformations including active, latent, and cleaved [54,62]. Unless bound to vitronectin, the active form can be readily converted to the more energetically favorable inactive latent form by internalizing the RCL, which may serve as a regulatory mechanism to prevent excessive anti-fibrinolysis [63,64,65]. However, the latent form can be reactivated. In its cleaved form, PAI-1 is still able to bind to other proteins with its helix, but its ability to inhibit uPA/tPA is abrogated [63]. As alluded to earlier, PAI-1 is a master regulator of the plasminogen system. PAI-1 can rapidly inactivate uPA/tPA with a second-order-rate constant between 106 and 107 m^−1^ s^−1^, forming a non-covalent Michaelis-like complex and eventually forming an ester bond between the serine residue of the protease and the carboxyl group of the P1 residue of PAI-1 [66,67]. PAI-1 also plays an important role in extracellular matrix (ECM) remodeling by indirectly modulating the activity of matrix metalloproteinases (MMPs) [68]. Indeed, by inhibiting the plasmin activation required for the cleavage of pro-MMP, PAI-1 can block ECM degradation [55]. 

## 3. Mechanisms Involved in PAI-1 Upregulation

**Figure 1:**
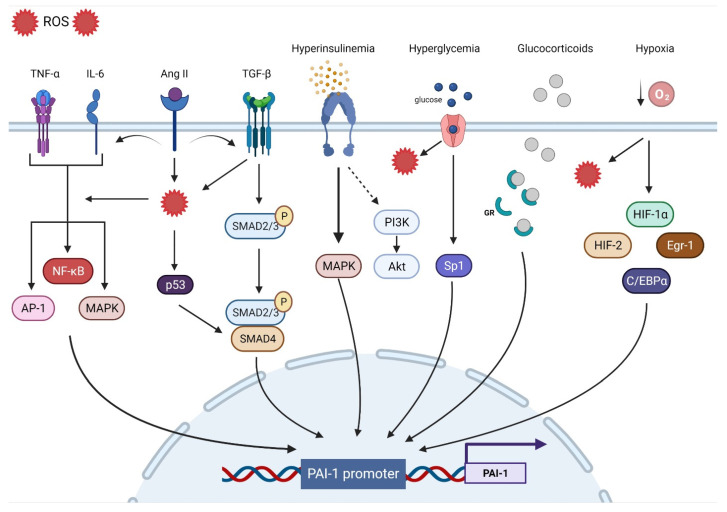
Transcriptional regulatory pathways implicated in PAI-1 synthesis.

The human PAI-1 promoter shows a high degree of homology with mice and rats, suggesting that they are regulated by similar mechanisms. The 5′-flanking region contains a ‘TATA’ box with several transcription binding sites including hypoxia inducible factor-1α (HIF-1α), Smads, activator protein-1 (AP-1), specificity protein-1 (SP-1), and nuclear factor kappa B (NF-ƘB). In the next section, we will discuss the major contributors to PAI-1 upregulation (Figure 1). 

### 3.1. Oxidative Stress

Oxidative stress (OS) is the end result of an imbalance between the production of oxidants and the capacity of the antioxidant system. Although they play an important role in regulating cellular function and signal transduction, free radicals such as reactive oxygen species (ROS) can be detrimental when produced in excess, given their ability to damage lipids, proteins, and DNA [69]. OS is undeniably a major contributor to multiorgan dysfunction in many disease states including CVD [70]. Indeed, ROS overproduction directly decreases nitric oxide (NO) bioavailability, uncouples eNOS, oxidizes low-density lipoprotein (OxLDL), and induces vascular inflammation [71]. OS is a significant upregulator of PAI-1 transcription. Indeed, incubating endothelial cells with H_2_O_2_ induced marked increases in PAI-1 mRNA and protein expression [72]. Conversely, the PAI-1 promoter was suppressed by up to 75% in the presence of antioxidants [73]. Furthermore, inhibiting NADPH oxidase, a major source of ROS, abolished the PAI-1 release and promoter activity in cultured endothelial cells [74]. Other experimental in vitro and in vivo studies performed in animal models as well as in humans have shown that the administration of antioxidants can decrease PAI-1 expression [20,75,76,77,78,79,80,81,82,83]. Due to their intricate interactions with multiple signaling pathways and transcription factors, ROS are involved in most of the mechanisms regulating PAI-1 expression. For instance, ROS-induced PAI-1 increased transcription and expression is mediated through the activation of mitogen-activated protein kinase (MAPK) and NF-ƘB pathways that are tightly involved in pro-fibrotic and pro-inflammatory pathways [74,84]. ROS signaling can also stimulate AP-1, HIF-1α, and p53, all of which can increase the transcription of PAI-1 [85,86] (Figure 1).

### 3.2. Inflammation

Inflammation is a complex constellation of reactions between the host normal defense processes to internal and external stressors that have been implicated in many conditions and age-related diseases, especially in promoting atherosclerosis, a hallmark of CVD [87,88,89]. Low-grade inflammation induces endothelial dysfunction and subintimal cholesterol accumulation, leading to the upregulation of intercellular adhesion molecules and selectins that promote the binding and transmigration of inflammatory cells including monocytes and T-helper cells into the vessel wall. Infiltrating monocytes can transform into resident macrophages that express and activate inflammasomes that are key to the propagation of inflammation through the generation of multiple cytokines that amplify the inflammatory cascade within the vessel wall [89]. Coupled with enhanced ROS production, inflammation enters a vicious cycle in combination with OS, further aggravating atherosclerosis [90]. The link between inflammation and the fibrinolytic system is well-established. Experimental in vitro and in vivo studies as well as clinical studies have identified tumor necrosis factor-α (TNF-α) as a substantial contributor to increased PAI-1 expression [91,92,93,94,95,96]. In endothelial cells, TNF-α upregulated PAI-1 levels and was abolished by N-acetyl cysteine, indicating ROS as a mediator [73]. Administration of TNF-α in mice significantly increased the PAI-1 levels in adipose tissue, while obese mice treated with antibodies targeting TNF-α exhibited reduced plasma PAI-1 expression and adipose tissue-PAI-1 levels [97,98]. It is suggested that TNF-α can induce PAI-1 gene expression via redox-sensitive mechanisms triggering NF-ƘB translocation and interaction with a regulatory region that is present on the PAI-1 promoter [21,96]. These data showcase the interplay between inflammation and OS and their integral role in upregulating PAi-1. Other pathways have been suggested in TNF-α-mediated PAI-1 induction including MAPK and protein kinase C [93]. Interleukin-6 (IL-6) is another inflammatory cytokine involved in PAI-1 upregulation. IL-6 is an acute phase inflammatory reaction protein that can induce C-reactive protein (CRP) synthesis and cortisol production [99]. Animals injected with IL-6 had significant increases in PAI-1 levels, while using IL-6 receptor antagonist reduced the PAI-1 expression in COVID patients [100,101]. IL-6 can activate NF-ƘB and MAPK, leading to increased PAI-1 transcription [55,102] (Figure 1). 

### 3.3. Fibrosis

Progressive vascular fibrosis is a prominent feature of atherosclerosis and CVD [103]. Transforming growth factor-β (TGF-β) is a major regulator of the fibroproliferative response to tissue damage [104]. TGF-β can control cell proliferation and migration, matrix synthesis, calcification, and immunomodulation, all being integral components of atherosclerosis [105]. TGF-β can be produced by all cells composing the vasculature and can also be produced in atherosclerotic lesions. However, TGF-β is mainly released by activated platelets adherent to activated endothelium. As a result, TGF-β induces the transcription of platelet-derived growth factor, collagens, fibronectin, and thrombospondins while suppressing the breakdown of ECM by inducing the transcription of PAI-1 and metalloprotease inhibitors, leading to the accumulation of the fibrotic matrix followed by calcification [103,105]. Overall, TGF-β production in atherosclerotic lesions can result in negative remodeling and progressive narrowing of the arteries, leading to MI and stroke [103]. TGF-β is considered as one of the major drivers of PAI-1 upregulation. In vitro studies have shown that PAI-1 expression is induced by TGF-β in various types of cells, while elevated PAI-1 levels are associated with enhanced TGF-β expression and ECM deposition under many pathological conditions [22,106,107,108,109,110,111]. TGF-β can induce PAI-1 production through the activation of the Smad pathway via the nuclear translocation of the Smad 2/3 and Smad 4 complex and binding to the PAI-1 promoter [112]. Interestingly, TGF-β can induce ROS production and suppress antioxidant activity in various types of cells and in vivo [113,114,115,116,117,118,119]. Thus, PAI-1 expression can also be mediated through TGF-β-induced ROS production. MAPK and NF-ƘB signaling are redox sensitive pathways that can be induced by TGF-β [55,114,120,121]. In TGF-β treated cells, inhibition of NADPH oxidase blocked TGF-β induced MAPK activated PAI-1 expression [85]. Furthermore, TGF-β can upregulate PAI-1 through Smad interactions with p53 and the transcription factors AP-1 and SP-1 [22,85,122] (Figure 1). 

### 3.4. Hypoxia

Hypoxia triggers many cellular processes both in physiological and pathological conditions and has been associated with vascular dysfunction and atherosclerosis [123]. Vascular wall cells respond to hypoxia by tuning metabolism, angiogenesis, inflammation, cell survival signaling, and ultimately, may develop endothelial dysfunction [124,125]. The main regulator of such processes is the transcription factor HIF-1α. Under normoxic conditions, HIF-1α is constantly degraded, whereas hypoxia promotes its stability and transcriptional activity [126]. However, HIF-1α is stabilized in atherosclerotic lesions even under normoxic conditions. ROS, OxLDL, NF-ƘB, and other factors are promoted by HIF-1α and in return, enhance HIF-1α stability [123]. PAI-1 is one of the main transcriptional targets of HIF-1α. Indeed, cells exposed to hypoxia display increased PAI-1 mRNA expression and stability [127,128,129,130,131]. HIF-1α knockdown limited irradiation-induced PAI-1 upregulation in endothelial cells [132]. ROS production in endothelial cells induced HIF-1α and subsequently PAI-1 production [133,134]. Additionally, ROS induced HIF-1α via a specific NF-ƘB binding site in the HIF-1 promoter [135]. Indeed, upregulation of the pulmonary artery smooth muscle PAI-1 was induced by an NF-ƘB-dependent HIF-1α transcription [136]. Although HIF-1α appears to dominate the PAI-1 transcriptional response to hypoxia, other pathways including HIF-2α, early growth response protein-1 (Egr-1), and CCAAT-enhancer-binding protein-α (C/EBPα) can augment this response independently of HIF-1α [137,138] (Figure 1). 

### 3.5. Hormones

Insulin can directly stimulate PAI-1 production in hepatocytes, an effect that is augmented by the presence of insulin-like growth factor [139,140]. The same effect was observed in cocultured endothelial cells and smooth muscle cells (SMCs) [141]. In the context of insulin resistance, compensatory hyperinsulinemia decreases the activity of the PI3-K/Akt pathway and augments the MAPK/ERK pathway, a major driver of PAI-1 production [142,143]. Elevated levels of glucose can also directly increase the expression of PAI-1 in endothelial cells and SMC through an effect on two adjacent Sp1 sites [122]. These data explain the elevated levels of PAI-1 in conditions characterized by hyperinsulinemia and hyperglycemia such as obesity, metabolic syndrome, and type 2 diabetes mellitus [25,144,145]. Under intense stress, very high levels of glucocorticoid hormones can increase the production of PAI-1 protein [146]. Glucocorticoids bind to their cytoplasmic glucocorticoid receptor and the complex is translocated to the nucleus and directly binds to the glucocorticoid response element that enhances PAI-1 transcription [86]. Angiotensin II, a major vasoconstrictor and contributor to hypertension upregulated by the activation of the renin–angiotensin–aldosterone system (RAAS), has been reported to induce PAI-1 expression in cultured endothelial cells in an angiotensin receptor independent manner [147]. Ang II can increase ROS production, fibrotic signaling (TGF-β), and inflammation, all of which can increase the expression of PAI-1 [148,149,150] (Figure 1). 

## 4. Pathological Role PAI-1 Role in Cardiovascular Disease

In humans, PAI-1 deficiency is a rare disorder that is attributed to mutations in the SERPINE1 gene that leads to either the absence of PAI-1 plasma detectable levels or the production of a non-functional PAI-1 protein [151,152,153]. The disease is characterized mainly by delayed mild to moderate bleeding following a traumatic event or injury, or during surgeries and in the contest of pregnancy complications [154,155]. Difficulty in establishing an accurate diagnosis stems from the fact that the PAI-1 activity assay detects elevated levels but is much less performant at the lowest detectable ranges [155]. Thus, the true prevalence of this rare condition is not well-established. On the other hand, two frequent PAI-1 gene polymorphisms have been shown to affect the PAI-1 levels [156,157]. The 4G/5G polymorphism that refers to single guanosine insertion/deletion at the transcription site is associated with higher PAI-1 activity, and the G/A polymorphism that refers to the single nucleotide substitution of guanine with adenine upstream of the transcription site leads to increases in the transcription rate [157,158]. Several clinical studies have suggested that PAI-1 polymorphisms (possibly leading to increased PAI-1 levels or activity) are an independent risk factor for major adverse cardiovascular events (MACE) including atherosclerosis, CAD, MI, stroke, and venous thrombosis [159,160,161,162,163,164,165,166]. Even in the absence of polymorphisms, elevated PAI-1 levels have been linked to the aforementioned events [23,167,168,169,170]. The Framingham Heart Study showed that PAI-1 levels are predictive of CVD events after accounting for established risk factors, while a serial increase in PAI-1 is associated with a further increase in risk [168]. Additionally, a recent meta-analysis identified 38 articles between 1991 and 2016 that reported PAI-1 levels in 11,557 patients. In studies assessing PAI-1 concentrations and activity levels, 15.1% and 29.6% of the patients included in these studies experienced MACE, respectively. Furthermore, patients with MACE had higher PAI-1 concentrations with a mean difference of 6.11 ng/mL [171]. However, not all studies confirmed a direct link between the elevated PAI-1 levels and CVD, especially after adjusting for the confounding factors [172,173,174,175]. It is very likely that the absence of such an association may be explained by the fact that factors such as age, sex, obesity, insulin resistance, and diabetes are positively correlated with plasma PAI-1 levels [25,175,176,177,178]. 

**Figure 2:**
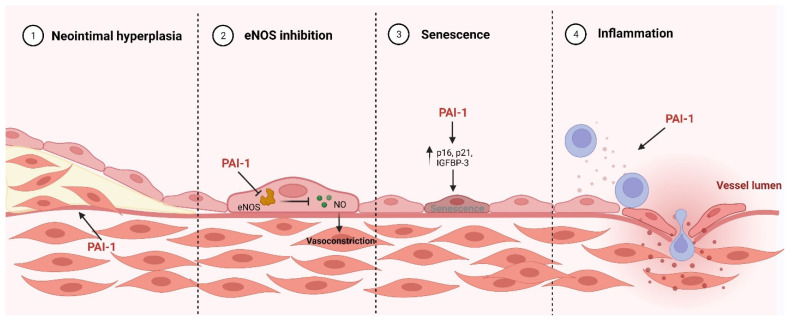
Pathological role of PAI-1 in the vasculature.

In order to comprehensively evaluate the pathological role of PAI-1, several mouse models have been developed. These murine lines are either completely PAI-1 deficient (PAI-1^−/−^) or overexpress native or stabilized human or murine PAI-1. PAI-1^−/−^ mice develop normally with no apparent macroscopic or microscopic histological abnormalities [179]. Although the deficiency of PAI-1 has been shown to increase the resistance to thrombosis and is protective against atherosclerosis [180,181,182], other studies have shown that the absence of PAI-1 can promote atherosclerosis and cardiac fibrosis [183,184,185]. It is suggested that abrogating the controlling effect of PAI-1 on the plasminogen system can contribute to the atherogenic and fibrotic role of plasmin, since the latter can mediate inflammation, foam cell formation, and ECM remodeling [186,187,188]. These data highlight the importance of the balance required between all the components of the fibrinolytic system to maintain homeostasis. For mice overexpressing PAI-1, transgenic mice overexpressing a stable active form of human PAI-1 (PAI-1 stab) display phenotypic abnormalities including alopecia and hepatosplenomegaly with age-dependent coronary arterial thrombosis, even in the absence of severe hypercholesterolemia [189,190]. In addition, transgenic mice overexpressing native human PAI-1 develop venous, but not arterial thrombosis [191]. For transgenic mice overexpressing stable murine PAI-1, they appear to suffer from an occasional tail autoamputation with no evidence of thrombosis [191]. The phenotypic differences observed could be attributable to cross-species differences and to the nature of the stable variant [54]. Although the major vascular pathological role of PAI-1 is related to its ability to create a hypofibrinolytic environment, the function of the PAI-1 extends beyond controlling fibrinolysis through the inhibition of plasmin formation as plasmin is involved in other physiological processes including ECM remodeling, angiogenesis, cell growth, and differentiation [192]. PAI-1 can also affect cell migration and signaling through the interaction with vitronectin and LDL receptor related protein 1 (LRP1). Several studies have noted additional anti-fibrinolytic independent mechanisms by which PAI-1 can induce endothelial dysfunction and atherosclerosis (Figure 2).

### 4.1. Pro-Inflammatory

As mentioned before, proinflammatory cytokines such as TNF-α and IL-6 can upregulate PAI-1 expression [93,100]. However, PAI-1 possesses the intrinsic ability to modulate inflammation. In alveolar epithelial cells stimulated by cigarette smoke extraction and lipopolysaccharides (LPS), expression of inflammatory factors and monocyte migration were detected. After transfection with siRNA-targeted PAI-1, these inflammatory indicators were attenuated, suggesting a proinflammatory role of PAI-1 at least in chronic obstructive pulmonary disease (COPD) [193]. Moreover, PAI-1 can modulate inflammation and induce macrophage infiltration in murine lungs after LPS-infusion through toll-like Receptor-4 (TLR4) [194]. More recently, it has been shown that PAI-1 promotes neutrophil diapedesis and tissue injury after ischemia-reperfusion (I/R). After I/R, PAI-1 accumulates on the endothelial cell surface and encounters rolling neutrophils expressing LRP1. PAI-1 then facilitates the adhesion of neutrophils through the intracellular adhesion molecule-1 (ICAM-1) triggering endothelial permeability, transmigration of neutrophils to the subendothelium, and ultimately inflammation and vascular injury [29] (Figure 2). Although the proinflammatory roles of PAI-1 have not been extensively studied in the setting of endothelial dysfunction, the few studies described earlier support the assumption that such effects may be involved in PAI-1-induced CVD. 

### 4.2. eNOS Inhibition

NO is a gaseous molecule that is synthesized by nitric oxide synthases from L-arginine with a half-life of 2–30 s [195]. In the endothelium, eNOS is the major producer of NO that diffuses to the smooth muscle cells and stimulates soluble guanylate cyclase, thereby relaxing SMCs and initiating vasodilation [195]. NO has also anti-thrombotic, antiproliferative, and anti-inflammatory properties [196]. An imbalance in NO production or in its bioavailability can induce endothelial dysfunction and subsequent CVD [197,198]. Several protein–protein interactions have been shown to modulate eNOS activity such as caveolin-1, heat shock protein 90, and hemoglobin-α [199]. Very recently, it has been uncovered that PAI-1 can be endocytosed by endothelial cells and directly bind to and suppress the ability of eNOS to produce NO [30] (Figure 2). Additionally, chemical inhibition of PAI-1 was shown to impair its interaction with eNOS and to enhance endothelium-dependent vasodilation in blood vessels [30]. Another recent study showed that delivery of recombinant PAI-1 to carotid arteries resulted in reductions in NO signaling and the enhancement of endothelial-derived hyperpolarization signaling [200]. This evidence incriminates PAI-1 as a direct mediator of endothelial dysfunction. 

### 4.3. Senescence

Senescence is an orchestrated cellular process characterized by the permanent termination of cellular proliferation. Tissue resident cells exhibit hallmarks of the cellular senescent phenotype predominantly during the development of age-related disorders including atherosclerosis [201]. Stress-induced premature cellular senescence is the major contributor to age-dependent vascular pathologies [202]. Quintessential senescent stimuli include ROS-mediated DNA damage, telomere erosion, and the activation of certain transforming genes [203,204]. Still, senescent cells are metabolically active and capable of producing factors called the senescence messaging secretome (SMS). Extensive evidence has identified PAI-1 as a prominent member of the SMS [28,205]. PAI-1 levels increase with age in many different tissues, which are associated with the increased incidence of stress-induced thrombosis in aged mice [206]. In a murine model of thrombosis, plasma PAI-1 levels were elevated in old thrombosed mice when compared to age-matched non-thrombosed mice or younger thrombosed mice [207]. These results indicate that the elevation of PAI-1 with age could predict the onset and progression of atherothrombosis in the elderly population. In endothelial cells, the majority of high passage cells were senescent and had upregulated levels of PAI-1, p21, and monocyte adhesion molecule, while the overexpression of SIRT-1 prevented stress-induced senescence by suppressing the PAI-1 levels and enhancing eNOS expression [208]. Several other in vitro studies showed that TGF-β and p53 pathways elevated PAI-1 levels and inhibited the proliferation of fibroblasts and keratinocytes. However, with the absence of PAI-1, TGF-β and p53 were unable to inhibit proliferation in both cells [209,210]. More importantly, overexpressing PAI-1 was sufficient to promote replicative senescence in fibroblasts [209]. These data strongly indicate that PAI-1 is not only a marker, but also a *bona fide* mediator of senescence. To confirm that PAI-1 induces vascular senescence in vivo, experiments using the inhibition of PAI-1 have been shown to reduce p16 levels and telomere attrition induced by eNOS inhibition in murine aortic tissue [211]. Additionally, in a murine model of accelerated aging (klotho hypomorph), plasma levels of PAI-1 were 45-fold higher than in wild-type mice with increased renal expression of p16 that was reduced after PAI-1 pharmacological inhibition with a noticeable increase in life span [212]. The mechanisms involved in PAI-1-mediated senescence are still unclear. One suggested pathway was the inhibition of insulin-like growth factor binding protein-3 (IGFBP-3) degradation. IGFBP-3 has been shown to directly induce cellular senescence and its depletion was protective against doxorubicin-induced senescence [213]. PAI-1 inhibition also decreased IGFBP-3, p21, p16, and p53 levels in doxorubicin-treated endothelial cells, fibroblasts, and cardiomyocytes [214] (Figure 2). Overall, it is evident that the PAI-1 plays an important role in mediating and controlling cellular senescence. 

### 4.4. Neointimal Hyperplasia

Neointimal hyperplasia is a prominent process involved in CVD such as atherosclerosis and restenosis after balloon angioplasty. Migration of SMCs from the media through the ECM into the intima is a key step in neointimal hyperplasia [215]. PAI-1 levels have been shown to increase in human vascular diseases characterized by neointima formation [216,217]. Through its interactions with vitronectin and LRP1, PAI-1 can mediate SMC adhesion and migration. PAI-1 binding to vitronectin inhibits its interactions with its receptors on SMC, thereby attenuating SMC adhesion and migration [218,219]. On the other hand, PAI-1 binding to LRP1 could promote SMC migration [220]. Thus, the concentrations of PAI-1 and vitronectin can influence neointimal formation. Pharmacological inhibition of PAI-1 in vitro and in vivo can prevent SMC migration and neointimal hyperplasia [31,221]. Indeed, targeting PAI-1 inhibited SMC migration through collagen gels including those supplemented with vitronectin, but did not inhibit the migration in endothelial cells and PAI-1 deficient SMCs [31]. Moreover, PAI-1 inhibition decreased the LRP-mediated signal transduction in SMCs that was markedly lower in endothelial cells. Importantly, targeting PAI-1 blocked intimal hyperplasia and inflammation in murine models of pathological vascular remodeling, but did not impair reendothelialization after mechanical denudation of the vascular endothelium [31]. These findings suggest an important role of PAI-1 in neointima formation, at least in settings involving atherosclerosis and restenosis (Figure 2). 

## 5. Is PAI-1 a Mediator of OSA-Induced CVD?

OSA is a chronic condition that is highly prevalent globally, especially among obese subjects. Extensive evidence links OSA to increased risk of CVD and overall mortality. The prevalence of OSA among stroke patients is estimated to be 50–70% [222], while up to 65% of patients who seek medical attention for a cardiovascular event are diagnosed with OSA [223]. Despite its high prevalence in patients with CVD and the susceptibility of cardiac patients to OSA-related stressors and adverse cardiovascular outcomes, OSA often remains under-recognized in the field of cardiovascular medicine. During sleep, OSA triggers IH coupled with sleep fragmentation that can induce elevations in blood pressure, OS, and inflammation [3,5,224]. Using rodent models of IH, hemodynamic changes emerge and lead to blood pressure alterations, along with impairments in vascular reactivity, ROS production, activation of proinflammatory cytokines, and altered lipid metabolism, all of which are important factors promoting endothelial dysfunction and atherosclerosis [4,5]. Unfortunately, the beneficial effects of current OSA therapies such as continuous positive airway pressure (CPAP) on CVD outcomes are inconsistent and fraught with scientific controversy. For instance, the largest randomized control study to date (SAVE) failed to demonstrate conclusive evidence of significant reductions in the primary end point (composite CVD) among patients treated with CPAP after a mean of 3.7 years follow-up [44]. A similar randomized clinical trial involving approximately 2500 subjects failed to identify OSA as an independent factor increasing the prevalence of ischemic coronary events, whereas treatment with CPAP did not significantly reduce the CAD prevalence [43]. Moreover, although incident CAD events are significantly enhanced by OSA, this risk is apparent only in those patients without a previous history of CAD [225]. This suggests that once the atherosclerotic vascular pathological processes reach more advanced stages, their reversibility with OSA treatment may not be possible, a finding that was recapitulated in mice exposed to IH for prolonged periods of time [226]. Furthermore, differential sex-specific responses to CPAP for OSA, at least for circulating inflammatory biomarkers even after adjusting for confounding factors, warrant further investigation to inform sex-specific personalized treatment approaches [227]. Ultimately, the need for additional adjuvant therapies aimed at the cardiovascular disturbances induced by OSA are needed.

**Figure 3:**
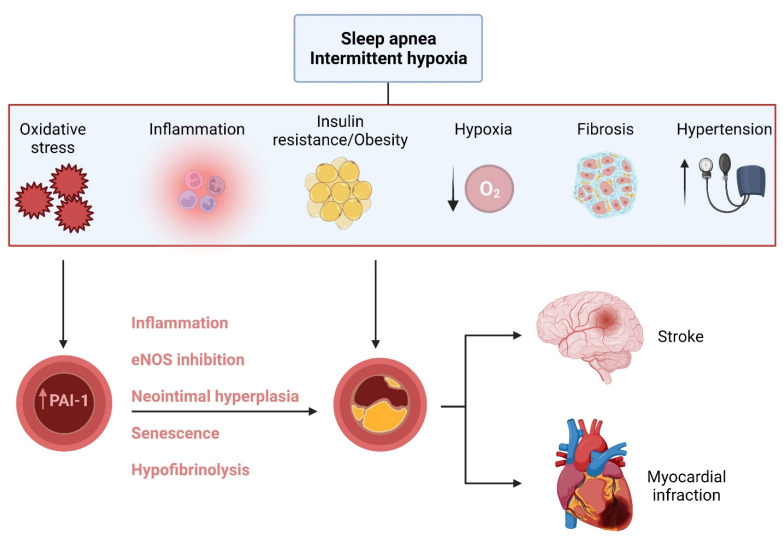
Putative role of PAI-1 in OSA induced CVD.

Circulating PAI-1 levels are elevated in OSA patients [32,33,34,35,36,37,38,39,40,41,42]. Indeed, OSA has been associated with a hypercoagulable state and a decrease in fibrinolytic activity [228], putting OSA patients at high risk of developing thrombosis [229,230,231]. As described earlier, ROS and proinflammatory cytokines are major drivers of PAI-1 transcription. Extensive evidence from clinical and experimental studies shows that lipid, protein, and DNA oxidative stress markers are all elevated in OSA patients and in animals exposed to IH [232,233,234,235,236,237,238,239]. Additionally, neutrophils and monocytes isolated from OSA patients were shown to be activated and exhibited increased ROS production [240,241]. Evidence from animals and cells exposed to IH also shows that NADPH oxidases, xanthine oxidase, and mitochondria are all major sources of ROS [224]. NF-ƘB has been shown to be activated in OSA and pro-inflammatory cytokines such as TNF-α, IL-6, and CRP are also all elevated in OSA patients [5,33,242,243,244,245]. Indeed, neutrophils from OSA patients showed an 8-fold greater NF-ƘB binding activity [246]. A recent meta-analysis identified a significant association between OSA and elevated TNF-α levels, while TNF-α levels were consistently correlated with the severity of OSA [247]. Furthermore, the hypoxic stimulus resulting from IH can promote HIF-1α signaling and contribute to the upregulation of PAI-1 [248]. Although clinical studies show normal or even reduced levels of plasma TGF-β levels (another major driver of PAI-1 transcription) in OSA [249,250], it has been shown that TGF-β increased with OSA severity in exhaled breath condensate, which can be normalized by CPAP treatment [250]. Furthermore, several animal studies have reported increased TGF-β/Smad signaling in renal, lung, and cardiac tissues when exposed to IH [251,252,253]. The majority of OSA patients have other or more coexisting co-morbidities including obesity, hypertension, diabetes, and metabolic syndrome [254,255,256,257]. Thus, the increased RAAS activation and the enhanced levels of Ang II, along with dyslipidemia, hyperglycemia, and insulin resistance may impose a synergistic effect on PAI-1 levels in OSA patients. Collectively, OSA appears to positively affect the PAI-1 levels as the majority of the mechanisms involved in PAI-1 upregulation can be triggered by OSA (Figure 3). 

As indicated in the aforementioned paragraphs, PAI-1 contributes to endothelial dysfunction and atherosclerosis through inflammation, decreased eNOS function, neointimal formation, and vascular senescence, all of which have been reported in OSA and animals exposed to IH (Figure 3). Impaired endothelial function has been reported in both children and adult patients with OSA [258,259]. In animals, a recent meta-analysis analyzed over 125 studies evaluating the impact of IH on vascular function reported that IH altered vasodilation and induced increases in vasoconstrictive responses [260]. Several other studies have reported that IH can uncouple vascular eNOS, reduce eNOS phosphorylation, or directly reduce NO bioavailability [7,8,261,262,263]. However, no studies have examined the potential inhibitory effects of PAI-1 on eNOS under IH settings. A meta-analysis of 18 studies confirmed that OSA is an independent risk factor for carotid intima media thickness (cIMT), even after adjusting for confounding factors [264]. Another meta-analysis in animals showed that cIMT significantly increases upon IH exposure and that IH increased atherosclerotic plaque size in ApoE ^−/−^ mice [260]. OSA is considered as an acceleration trigger of cellular senescence. Indeed, it has been suggested that OSA can cause telomere shortening through enhanced oxidative stress, hypoxia, inflammation, and circadian clock disturbances [265]. Recently, plasma exosomes isolated from untreated OSA patients were shown to increase the senescence markers of naïve endothelial cells including p16 and x-gal, while similar cells exposed to IH recapitulated the same senescent phenotype [266]. Furthermore, accelerated epigenetic age clock was detected in patients with OSA when compared to the matched controls, and furthermore, adherent treatment with CPAP resulted in the deceleration of epigenetic aging [267]. However, the role of PAI-1 in promoting neointimal formation and mediating vascular senescence has yet to be evaluated in OSA. Thus, it is plausible that OSA-induced vascular dysfunction can be mediated, at least in part, by deregulated PAI-1-related pathways (Figure 3). Future experimental studies assessing the impact of IH in vitro and transgenic mouse lines of PAI-1 will provide valuable insights into the mechanisms by which PAI-1 induces vascular dysfunction in the context of OSA. 

Given that PAI-1 is an independent risk factor for MACE, that PAI-1 shows elevated levels in OSA patients, and that there is a failure of conventional treatments to prevent adverse cardiovascular outcomes in OSA patients, it is tempting to propose that targeting PAI-1 may be advantageous in OSA patients with a risk of CVD. Many approaches have been dedicated to the development of PAI-1 inhibitors including small molecules, synthetic peptides, RNA aptamers, and monoclonal antibodies. The mechanisms of action by which these inhibitors are operationally active include: (i) blocking the initial formation of the Michalis complex between PAs and PAI-1; (ii) accelerating the transformation of active PAI-1 to its latent inactive form; or (iii) impeding the formation of the final inhibitory complex, leading to the substrate behavior of PAi-1 [54]. Several experimental studies have shown that PAI-1 inhibitors can inhibit metabolic dysregulation, improve endothelial function, and prevent atherosclerosis in the setting of diet-induced obesity [31,211,268,269]. Despite the extensive characterization of PAI-1 inhibitors and the promising results from the in vitro and in vivo studies, none of the existing PAI-1 inhibitors have yet to be approved for use in humans. This is mainly due to the affinity and specificity issues, structural plasticity of PAI-1, and the counteraction of PAI-1 binding proteins that can modulate its activity (such as vitronectin) [54]. However, evaluating the potential beneficial effects of PAI-1 inhibitors in the setting of IH is essential to assess whether PAI-1 is potentially a recommended approach as a therapeutic target in OSA-mediated CVD. 

## 6. Conclusions

OSA is a chronic and extremely frequent condition that is associated with endothelial dysfunction, atherosclerosis, and overall cardiovascular risk and mortality. PAI-1 is a key regulator of the plasminogen system required for control fibrin stabilization to prevent bleeding. However, elevated levels of PAI-1 may increase the risk of thrombosis and promote atherosclerosis through antifibrinolytic-independent mechanisms. OSA can trigger several signaling pathways involved in enhancing PAI-1 transcription. Thus, being elevated in OSA patients, PAI-1 could play an additive role in OSA-induced CVD. However, PAI-1 influence on CVD in the setting of OSA has yet to be addressed. To this effect, experimental studies evaluating the impact of IH in PAI-1 deficient, overexpressing, and vascular-specific deletion transgenic animals are critically needed to elucidate the role of PAI-1 in OSA-induced CVD. Furthermore, the use of PAI-1 inhibitors under IH conditions may also provide insights into the effectiveness of PAI-1 antagonism in preventing or mitigating OSA-mediated CVD. Therefore, PAI-1 could spark clinical interest as a putative drug target for the treatment of PAI-related CVD in OSA. 

## Data Availability

Not applicable.

## Abbreviations

AP1	Activation protein 1
CAD	Coronary artery disease
CPAP	Continuous positive airway pressure
CRP	C-reactive protein
CVD	Cardiovascular disease
ECM	Extracellular matrix
eNOS	Endothelial nitric oxide synthase
GR	Glucocorticoid receptor
HIF-1α	Hypoxia-inducing factor-1α
IH	Intermittent hypoxia
IL-6	Interleukin-6
LRP1	Low density lipoprotein receptor-related protein 1
MACE	Major adverse cardiovascular events
MAPK	Mitogen-activated protein kinase
MI	Myocardial infarction
MMP	Matrix metalloproteinase
NF-κB	Nuclear factor kappa B
OSA	Obstructive sleep apnea
PAI-1	Plasminogen activator inhibitor-1
RCL	Reactive center loop
ROS	Reactive oxygen species
SMC	Smooth muscle cell
Sp1	Specificity protein 1
TGF-β	Transforming growth factor-β
TNF-α	Tumor necrosis factor
tPA	Tissue-type plasminogen activator
uPA	Urokinase-type plasminogen activator
uPAR	Urokinase-type plasminogen activator receptor
